# Babies before bottom lines: A call for Australia to end exploitative marketing of commercial milk formula at home and abroad

**DOI:** 10.1016/j.lanwpc.2022.100640

**Published:** 2022-11-14

**Authors:** Madeleine Munzer, Jennifer Cashin, Nicole Jameson, Constance Ching, Sedtha Chin, Kroeun Hou, Chan Myae Aung, Paul Zambrano, Duong Vu Hoang, Roger Mathisen

**Affiliations:** a Independent Breastfeeding Advocate, Sydney, Australia; b Alive & Thrive Southeast Asia, FHI Solutions, Washington, DC, USA; c Alive & Thrive Southeast Asia, FHI Solutions, Scaling Up Nutrition Civil Society Alliance in Cambodia, Phnom Penh, Cambodia; d Helen Keller International, Scaling Up Nutrition Civil Society Alliance in Cambodia, Phnom Penh, Cambodia; e Alive & Thrive Southeast Asia, FHI Solutions, Scaling Up Nutrition Civil Society Alliance Myanmar, Yangon, Myanmar; f Alive & Thrive Southeast Asia, FHI Solutions, Manila, Philippines; g Alive & Thrive Southeast Asia, FHI Solutions, Scaling Up Nutrition Civil Society Alliance Viet Nam, Hanoi, Viet Nam

Globally, 600,000 deaths among women and children each year are attributable to not breastfeeding.^1^^,^^2^ Breastfeeding is often perceived as less important in high-income countries; however, it is no less critical for infant food security and normal health, growth, and development.^3^ Infants who are not breastfed are more vulnerable to death and disease,^4^ and this vulnerability has been highlighted during recent natural disasters, the pandemic, and the United States infant death and commercial milk formula shortage.^5^

Breastmilk substitutes are a type of commercial milk formula marketed as partial or total replacements for breastmilk for children 0–36 months of age. Widespread marketing and promotion of commercial milk formula influences feeding decisions and negatively impacts breastfeeding rates.^6^ Australia is the world's sixth largest exporter of commercial milk formula and a major supplier to Southeast Asia and China.^7^ As part of public health policy, governments must restrict unethical corporate practices. The Australian Government has declined opportunities to correct historical inaction on the issue of unethical and exploitative marketing of commercial milk formula products^8^ and is instead supporting predatory commercial milk formula marketing strategies at home and abroad.^9^

The Code of Marketing of Breast-milk Substitutes and subsequent World Health Assembly resolutions (the Code) is a set of recommendations adopted by the World Health Assembly (WHA) to curb the harmful marketing of commercial milk formula linked to morbidity and mortality among children.^10^ The Code is not legally binding on its own, requiring national adoption into law and enforcement for it to be effective. A recent WHO report found that only 32 countries have legislation that substantially aligns with the Code.^11^ Australia was not among them.

Instead of binding legal measures, Australia has adopted a voluntary system of industry self-regulation among signatory companies, called the *Marketing in Australia of Infant Formulas: Manufacturers and Importers Agreement* (MAIF).^12^ MAIF is among the weakest measures globally.^11^ It relies on consumer reporting and does not sanction violations of the agreement. Significant concerns have also been raised about the independence and transparency of the complaints-handling process.^8^^,^^13^ In 2021, the Australian Competition and Consumer Commission (ACCC) considered the re-authorisation of the MAIF and, prior to its decision, welcomed submissions from stakeholders. The ACCC received local^14^ and international submissions, including from civil society in Myanmar^15^ and Cambodia,^16^ which provided evidence contrary to the claim by the commercial milk formula lobby that the MAIF contributes to public benefit.^17^ These submissions called for Australia to adopt binding national legislation on the Code and noted a wide range of international exploitative marketing activity by Australian commercial milk formula companies. Examples included violations of local legislation by Australian diplomats,^18^ promotion of commercial milk formula products within healthcare systems, celebrity endorsements, and misleading and inaccurate health claims.^15^^,^^16^

The ACCC re-authorised the self-regulatory, non-binding standards already shown to be ineffective in protecting public health^19^ despite expert and public opinion calling for change.^14^ However, it did so for a limited term of only three years to allow time for the Australian Commonwealth Department of Health (DoH) to conduct its own review of MAIF. Furthermore, ACCC noted that health policy issues are not a matter of competition law but the responsibility of the DoH and that the re-authorisation of MAIF does not represent an endorsement of the adequacy of the agreement.^8^ There will be significant local and international interest in the forthcoming review of the MAIF as an opportunity for Australia to legislate the Code and implement an alternate regulatory approach to adopt trade and export standards that align with the Code. At least 11 countries in the Asia Pacific region called for this during the 75th World Health Assembly in May 2022.^20^

Families worldwide have a right to make infant feeding decisions free from commercial influence. However, the commercial milk formula industry will not cede potential market share out of benevolence. To properly protect breastfeeding and prevent the unique vulnerability and food insecurity accompanying dependence on commercial milk formula, governments must impose legally enforceable limits on corporate marketing activity at all levels of policy and society. It has been over forty years since the WHA, including Australia, ratified the Code. Yet, Australia fails to enforce the Code at home and supports Australian companies in undermining Code legislation in neighbouring countries.^15^^,^^16^ It is time for Australia to correct historical inaction and end exploitative marketing at home and abroad.

## Contributors

Madeleine Munzer (M.M.) conceived and wrote the first draft of this commentary. M.M., Jennifer Cashin (J.C.), Nicole Jameson (N.J.), Constance Ching (C.C.), Sedtha Chin (S.C.), Kroeun Hou (K.H.), Chan Myae Aung (C.M.A.), Paul Zambrano (P.Z.), Duong Vu Hoang (D.V.H.), and Roger Mathisen (R.M.) revised and edited various drafts and provided documentation referenced in the commentary. All authors provided important intellectual contributions and ensured its final content.

## Declaration of interests

M.M. is a former Director of Breastfeeding Advocacy Australia (until June 2022). The Alive & Thrive initiative, managed by FHI Solutions, is currently funded by the Bill & Melinda Gates Foundation, the Government of Ireland, UNICEF, and the World Bank. Staff time to write this manuscript was covered by grants from the Bill & Melinda Gates Foundation (OPP50838) and the Government of Ireland to Alive & Thrive/FHI Solutions (C.C., S.C., C.M.A., P.Z., D.V.H., J.C., and R.M.). The views and opinions set out in this article represent those of the authors, and do not necessarily represent the position of the funders. All other authors have no conflict of interest to declare.
